# Modified Porous SiO_2_-Supported Cu_3_(BTC)_2_ Membrane with High Performance of Gas Separation

**DOI:** 10.3390/ma11071207

**Published:** 2018-07-13

**Authors:** Chunjing Lu, Gang Wang, Keliang Wang, Daizong Guo, Mingxing Bai, Ying Wang

**Affiliations:** 1 Key Laboratory for EOR Technology (Ministry of Education), Northeast Petroleum University, XuefuRoad 99, Daqing 163318, China; chunjinglu@outlook.com (C.L.); klwang0608@outlook.com (K.W.); baimingxing@hotmail.com (M.B.); 2 Mechanical Science and Engineering College, Northeast Petroleum University, XuefuRoad 99, Daqing 163318, China; daizongguo@outlook.com; 3 State Key Laboratory of Inorganic Synthesis & Preparative Chemistry, Jilin University, QianjinRoad 2699, Changchun 130012, China; ywang0707@outlook.com

**Keywords:** porous SiO_2_ disk, N-[3-(Trimethoxysilyl)propyl]ethylenediamine, modified, Cu_3_(BTC)_2_ membrane, gas separation

## Abstract

The structures and applications of metal-organic framework materials (MOFs) have been attracting great interest due to the wide variety of possible applications, for example, chemical sensing, separation, and catalysis. N-[3-(Trimethoxysilyl)propyl]ethylenediamine is grafted on a porous SiO_2_ disk to obtain a modified porous SiO_2_ disk. A large-scale, continuous, and compact Cu_3_(BTC)_2_ membrane is prepared based on a modified porous SiO_2_ disk. The chemical structure, surface morphology, thermal stability, mechanical stability, and gas separation performance of the obtained Cu_3_(BTC)_2_ membrane is analyzed and characterized by means of X-ray diffraction (XRD), scanning electron microscopy (SEM), thermogravimetric analysis (TGA) and a gas separation experiment. The results show that the prepared Cu_3_(BTC)_2_ membrane has an intact morphology with its crystal. It is continuous, compact, and intact, and has good thermal stability and mechanical stability. The result of the gas separation experiment shows that the Cu_3_(BTC)_2_ membrane has a good selectivity of hydrogen and can be used to recover and purify hydrogen.

## 1. Introduction

During the past few decades, the development of membrane materials has drawn research interests in both research-oriented and industrial applications [1,2,3]. The membrane is a barrier, which can selectively control some materials to pass through and make other substances not. As a result, the membrane can be used to separate mixtures [4,5,6]. Compared with traditional separation methods, the separation-based membrane has the advantage of energy saving and efficiency. Traditionally, the development and application of membranes involve documentation of the polymer membrane because it has disadvantages of short life, low working temperature, and lower chemical stability and selectivity, but researchers need to explore some new membrane materials that have stable performance and are more conducive to separation [7,8,9]. Metal-organic framework materials (MOFs) have become excellent candidates for membrane fabrication because they have diverse structures, uniform pore size, permanent porosity, and high thermal and chemical stability. The membrane separation is mainly based on the molecular-size sieve, which also can separate some materials that can react with the membrane through the method of adsorption and diffusion. The MOF membrane has developed quickly, because scientists can control the pore size easily by changing the metal ions and organic ligands while also modifying the surface of their pores through some approaches. Although more MOF membranes have been successfully synthesized, how to make the membrane have higher gas permeability as well as higher selectivity also offers more challenges [10,11,12].

A convenient, low-cost, and universal technique of preparing MOF membranes is essential for exploring the relationships between their structures and properties [13,14,15]. Metal-ligand coordination bonding interactions between the MOF membrane and substrate is the most widely used strategy to construct MOF composite membranes. To date, all reported methods of preparing MOF membranes have been limited to specific MOF membranes and specific surface-functionalized substrates, producing some limited methods due to the high cost [16,17,18,19]. Thus, far, there are few reports on the preparation of large-scale continuous compact MOF membranes with low cost.

Hydrogen is the most ideal source of energy known at present. It has many advantages, such as higher heat, wide sources, and no pollution of products. Its most attractive prospect is to replace fossil fuel as a power source for vehicles, which can significantly reduce the exhaust of air pollutants such as CO_2_, CO, NOx and more: removal at the source eliminates the greenhouse effect and haze threat to mankind and realizes the potential of low carbon and environmental protection [20,21]. However, due to its high production cost, difficultly of storage, transport complications and other shortcomings, its extensive popularization and application has been hindered. Presently, the main use of hydrogen is an important chemical raw material, such as ammonia gas and methanol [22,23]. The main sources of hydrogen used in the industry are coal (dry distillation, gasification of coke oven gas, gas), and petroleum or natural gas (converted to CO + H_2_ syngas) and other fossil fuels [24,25]. These methods obviously do not get pure hydrogen. During actual industrial production, to obtain high-purity hydrogen, the above hydrogen containing gas (CO + H_2_) is converted to an H_2_ + CO_2_ mixture first, and then the purity of hydrogen is higher than 99% by the method of pressure variable absorption and membrane separation [26,27]. The source of hydrogen is very wide, such as the gas in the ammonia plant, the by-product coke oven gas of the coking plant, the by-product hydrogen in the chlor alkali factory and more. Generally, they are discharged into the atmosphere as exhaust gases. This causes great waste and pollution. Science can take the appropriate method to reduce the production cost of hydrogen and promote the production cost of hydrogen [28,29,30]. Based on this background, the authors can adsorb and separate these mixtures (CO_2_, CH_4_ and H_2_) to recycle hydrogen. This study uses the Cu_3_(BTC)_2_ composite membrane to purify hydrogen, because Cu_3_(BTC)_2_ is a mature MOF, which has a regular pore structure, good thermal stability, and chemistry. The structures and applications of Cu_3_(BTC)_2_ membranes have been attracting great interest because of the wide variety of possible applications, for example, chemical sensing, separation, catalysis, and electromagnetism [31,32,33]. Concurrently, the SiO_2_ substrate has a high gas flux as a very good supporting role [34,35]. It is beneficial to separate and purify hydrogen and has a long service life. It is feasible for large-scale commercial applications.

The Cu_3_(BTC)_2_ membrane is synthesized to be used to separate and purify hydrogen on the SiO_2_ disk modified by N-[3-(Trimethoxysilyl)propyl]ethylenediamine. The method of preparing the MOF membrane or zeolite membrane using an organosiloxane agent modified base has been previously reported [36,37,38,39,40]. The XRD shows that the prepared Cu_3_(BTC)_2_ membrane has an intact morphology with its crystal, and the SEM shows that it is continuous, compact, and intact, while having a good thermal stability and mechanical stability. The separation factors of the Cu_3_(BTC)_2_ membrane for H_2_/CO_2_, H_2_/N_2_, H_2_/CH_4_ is 10.07, 10.20 and 11.34. The results show that Cu_3_(BTC)_2_ membrane has a good selectivity for hydrogen and can be used for recovery of hydrogen.

## 2. Materials and Methods

### 2.1. Materials

Porous SiO_2_ disks (diameter = 2.0 cm) were purchased from the Sinopharm Chemical Reagent Company (Shanghai, China). Ethanol (C_2_H_5_OH) and N-[3-(Trimethoxysilyl)propyl]ethylenediamine (C_8_H_22_N_2_O_3_Si), Cupric nitrate (Cu(NO_3_)_2_·3H_2_O) and trimesic acid (H_3_BTC) were purchased from Sigma Aldrich (St. Louis, MO, USA). All products were used as received.

### 2.2. Pretreatment of the SiO_2_ Disk

The porous SiO_2_ disk (diameter = 2.0 cm) was soaked into the mixed solution of the concentrated sulfuric acid and the hydrogen peroxide with the volume ratio of 6:4 for 5 h making the surface completely oxidized. To follow, the porous SiO_2_ disk was taken out and placed into the beaker of 50 mL. 30 mL of deionized water was added into the beaker. The solution in the beaker was conducted by ultrasonic treatment for 10 minutes and poured out. The washing process was repeated three times. The porous SiO_2_ disk was dried at 120 °C for 2 h.

### 2.3. Surface Modification of the SiO_2_ Disk with N-[3-(Trimethoxysilyl)propyl]ethylenediamine

N-[3-(Trimethoxysilyl)propyl]ethylenediamine (1 mL) and alcohol (50 mL) were added to the beaker and hydrolyzed for ten minutes. The oxidized porous SiO_2_ disk slice was placed horizontally at the bottom of the beaker and stirred for 24 h at 25 °C. Following the reaction, the SiO_2_ disk was washed with anhydrous ethanol repeatedly to remove the N-[3-(Trimethoxysilyl)propyl]ethylenediamine which was not functionalized. Then the functionalized porous SiO_2_ disk was dried in a vacuum.

### 2.4. Synthetic Cu_3_(BTC)_2_ Membrane with the Modified SiO_2_ Disk

**Figure 1:**
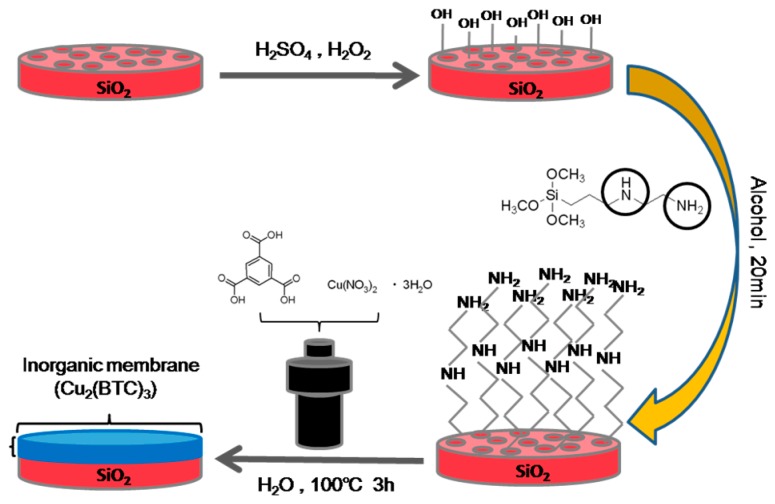
The process of preparing MOF membranes.

The MOF membranes were prepared by means of the hydrothermal method. The MOF membrane chosen was Cu_3_(BTC)_2_. Then, 0.7 g of cupric nitrate was dissolved in 19.2 mL of distilled water and solution A was obtained. Similarly, 0.336 g trimesic acid was dissolved in 19.2 mL ethanol and solution B was obtained. Solution B was then poured into solution A and stirred for 1 h. The mixed solution was poured into the Teflon-lined autoclave. The substrate of the functional porous SiO_2_ disk was placed in the Teflon-lined autoclave with tweezers at 100 °C for 3 d [41]. Then, the membrane was washed several times with ethanol and dried at 25 °C. The Cu_3_(BTC)_2_ crystals adhered to the membrane surface were washed away with ethanol and the dried MOF membrane based on the functional porous SiO_2_ disk was prepared by air-drying. A schematic diagram of the whole synthesis process is shown in Figure 1.

### 2.5. Characterization of the Cu_3_(BTC)_2_ Membranes

The thermogravimetric analysis (TGA) was performed using a DTG-60 thermal analyzer system (Shimadzu Corporation, Kyoto, Japan) at the heating rate of 10 °C min^−1^ to 900 °C in a dried air atmosphere. The air flow rate was 30 mL min^−1^. Samples were loaded in a platinum pan. The FTIR spectra (KBr Sigma, Aldrich, St. Louis, MO, USA) were measured using a IRAFFINITY-1 Fourier transform infrared spectrometer (Shimadzu Corporation, Nakagyo-ku, Kyoto, Japan). Samples were packed firmly to obtain transparent films. PXRD studies were performed using a D/MAX2550 diffractometer (Riguku Corporation, Akishima, Tokyo, Japan) using Cu-Ka radiation, 40 kV, 200 mA with a scanning rate of 0.3° min^−1^ (2θ). Scanning Electron Microscopy (SEM) analysis was performed on a JSM 6700 (JEOS Corporation, Akishima, Tokyo, Japan).

### 2.6. Low-Pressure N_2_ Sorption Measurements

Nitrogen sorption experiments were performed at 77 K up to 1 bar using a manometric sorption analyzer Autosorb iQ MP (Quantachrome Instruments, Boynton Beach, FL, USA). Prior to sorption analysis, the sample was evacuated at 150 °C for 10 h using a turbomolecular vacuum pump. Specific surface areas were calculated from nitrogen adsorption data by multipoint Brunauer-Emmett-Teller (BET) analysis. Pore size distributions were calculated from the N_2_ adsorption isotherms using a quenched solid density functional theory (nitrogen on carbon, slit pore) method which gave the least fitting error.

### 2.7. The Gas Separation Test

**Figure 2:**
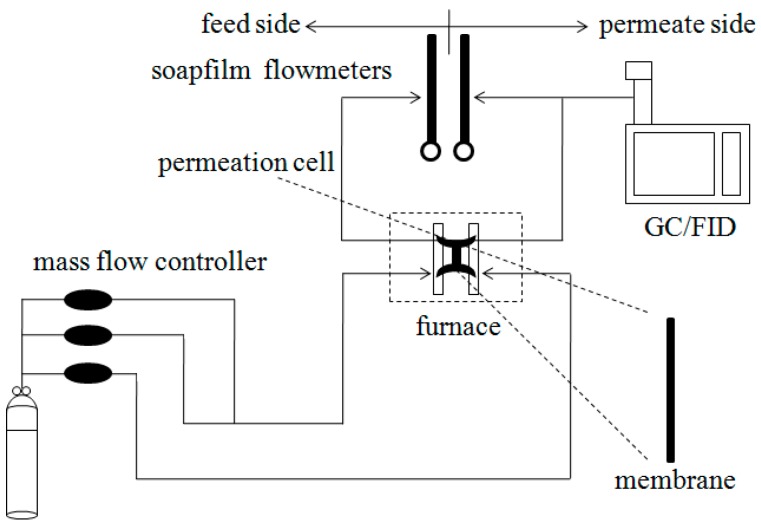
Schematic of gas separation process.

Prior to gas permeation measurements, the membranes were sealed in modules and swept by using Ar (sweep gas) and detecting gas. Meanwhile, the modules were heated to 80 °C and held for 1 h, then cooled to room temperature. Regarding both single component and mixture permeation, the permeate side and the feed side pressure were both set to 1 bar. Concerning mixture permeation, both feed gas and sweep gas rates were 80 mL min^−1^. A soap-film flow meter was used to measure the flux of the gas and the volume ratio of the mixture gas. This assembly is shown in Figure 2.

## 3. Results

### 3.1. The FTIR of the Modified SiO_2_ Disk

**Figure 3:**
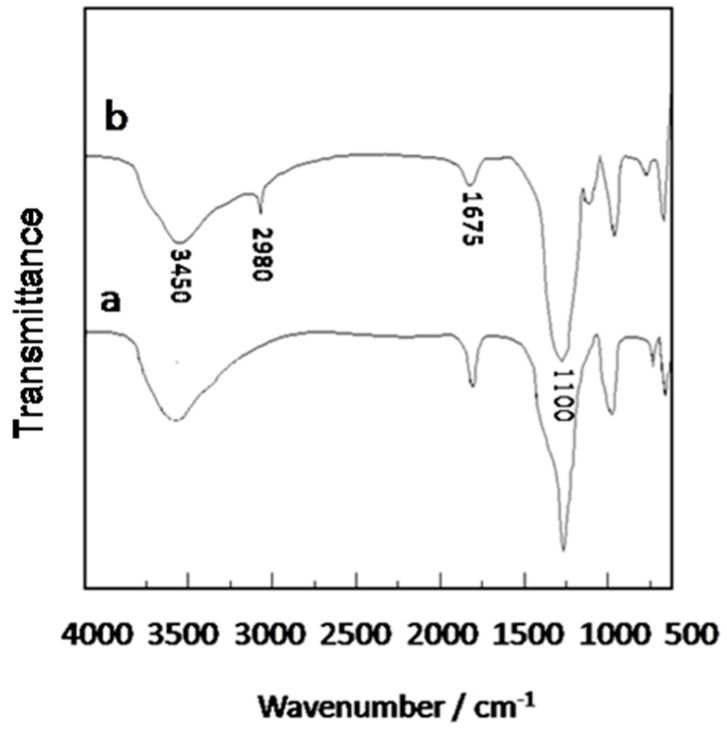
FTIR spectra of the porous SiO_2_ disk (a) and the modified porous SiO_2_ disk (b).

The FTIR spectra of the produced porous SiO_2_ disk and the modified porous SiO_2_ disk, demonstrated that the N-[3-(Trimethoxysilyl)propyl]ethylenediamine groups were grafted onto the porous SiO_2_ disk surface, as presented in Figure 3. Regarding the case of the porous SiO_2_ disk, the sharp band at 3450 cm^−1^ corresponded to the presence of silanol groups (Si–OH) on the silica surface. The absorption bands at 1645 cm and 1080 cm^−1^ were related to the bending vibration of H_2_O and the isolated terminal silanol (Si–OH) groups, respectively. Following modification with N-[3-(Trimethoxysilyl)propyl]ethylenediamine, the absorption of water and the Si–OH absorption peak intensity decreased, which was due to the surface of the porous SiO_2_ hydroxyl (–OH) and N-[3-(Trimethoxysilyl)propyl]ethylenediamine condensation reaction reducing the number. This changed the degree of bonding of the porous SiO_2_ surface to water, that is, the bonding density with hydrogen to produce hydrogen changes. The characteristic absorption peak after N-[3-(Trimethoxysilyl)propyl]ethylenediamine appeared at 2980 cm^−1^ due to the asymmetric stretching of the C–H bond in the aminopropyl group, indicating that N-[3-(Trimethoxysilyl)propyl]ethylenediamine had been grafted onto the porous SiO_2_ surface.

### 3.2. The XRD of the Cu_3_(BTC)_2_ Membranes

**Figure 4:**
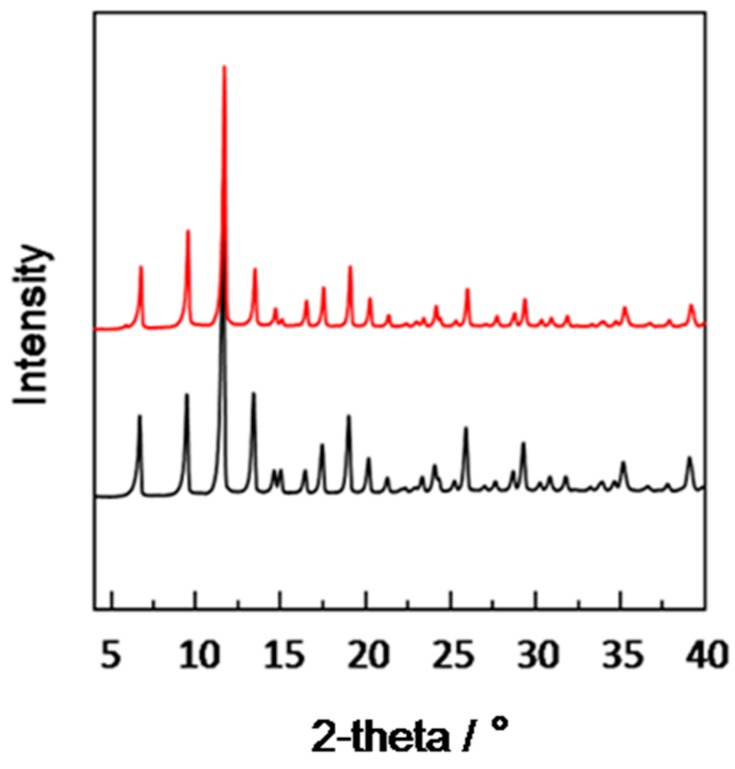
XRD spectra of Cu_3_(BTC)_2_ powder (red) and the prepared Cu_3_(BTC)_2_ membrane (black).

Figure 4 is the XRD spectrum of the modified porous SiO_2_ disk-supported Cu_3_(BTC)_2_ membrane (black) and the Cu_3_(BTC)_2_ powder (red). Figure 4 shows the apex position of the XRD peak of the Cu_3_(BTC)_2_ membrane was the same as the highest position of the XRD spectrum of the Cu_3_(BTC)_2_ powder. The phenomenon illustrates that the modified porous SiO_2_ disk-supported Cu_3_(BTC)_2_ membrane is a pure phase composed of Cu_3_(BTC)_2_ crystal.

### 3.3. The TGA of the Cu_3_(BTC)_2_ Membranes

**Figure 5:**
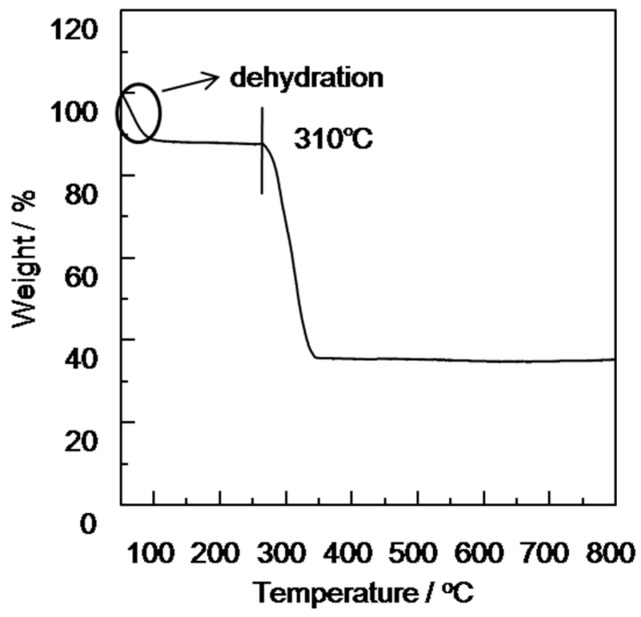
TGA curve of the modified porous SiO_2_ disk-supported Cu_3_(BTC)_2_ membrane.

The TGA was conducted to investigate the thermal stability of the modified porous SiO_2_ disk-supported Cu_3_(BTC)_2_ membrane. The results illustrate that the modified porous SiO_2_ disk-supported Cu_3_(BTC)_2_ membrane, at 63 °C, had a weight loss of 5%, which was the adsorbed water, and the Cu_3_(BTC)_2_ membrane was stable in the air to 310 °C, showing its good thermal stability. The thermogravimetric curve is shown in Figure 5.

### 3.4. The Low-Pressure N_2_ Sorption Measurements and the Pore Size of the Cu_3_(BTC)_2_ Membranes

**Figure 6:**
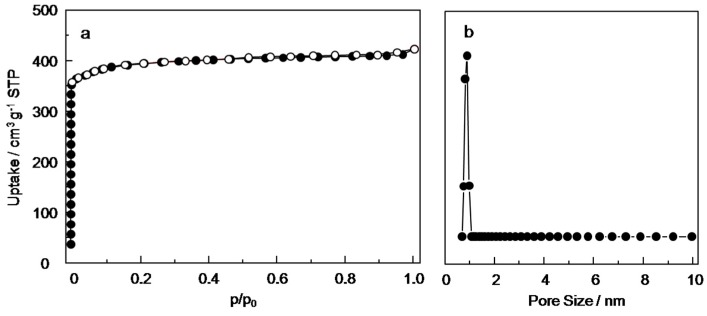
N_2_ sorption isotherms (**a**) and the pore size (**b**) of Cu_3_(BTC)_2_ membrane.

The low-pressure N_2_ sorption measurements and the pore size of the Cu_3_(BTC)_2_ membranes were revealed by nitrogen sorption isotherm measurement at 77 K (Figure 6). The samples both were activated and degassed 10 h at 150 °C and measured from 0 to 1 bar (1 bar = P_0_). The result of the Cu_3_(BTC)_2_ membranes exhibited a type I isotherm, which is a typical feature of microporous materials. The BET surface area was evaluated, and pore diameter was consistent with those previously reported, indicating that the m as 1550 m^2^ g^−1^. The pore size was calculated by appropriate fitting of the density functional theory model to the isotherm yields, which was a value of 1.0 nm for the Cu_3_(BTC)_2_ membrane. BET surface area and pore diameter were consistent with those previously reported, indicating that the membrane material had the same adsorption performance as the powder material [42].

### 3.5. The SEM of the Cu_3_(BTC)_2_ Membranes

**Figure 7:**
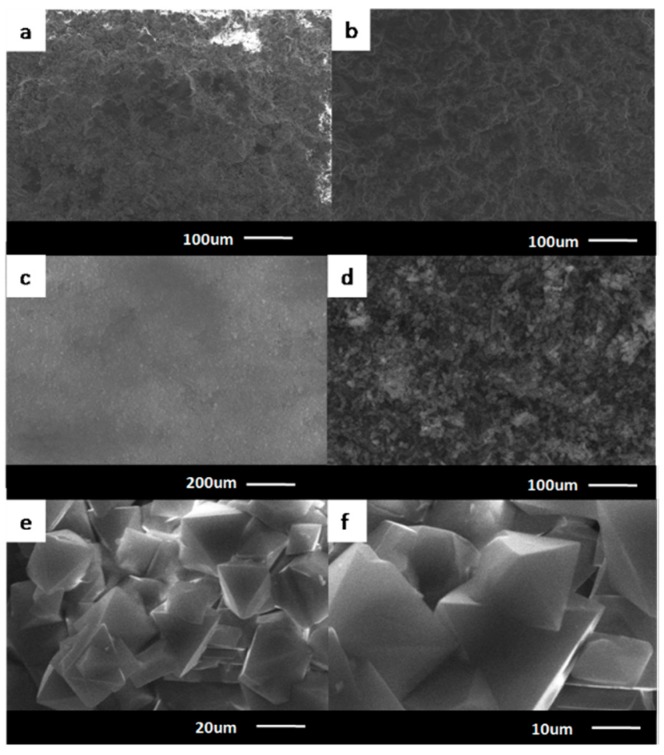
SEM of the prepared samples, with porous SiO_2_ disk (**a**), the modified porous SiO_2_ disk (**b**) and the modified porous SiO_2_ disk-supported Cu_3_(BTC)_2_ membrane (**c**–**f**).

The characterizations of the morphology of before and after the SiO_2_ disk were modified and the modified porous SiO_2_ disk supporting the Cu_3_(BTC)_2_ membrane was conducted after defining the structural information and thermal stability of this membrane. The SEM of the modified porous SiO_2_ disk-supported Cu_3_(BTC)_2_ membrane is shown in Figure 7. It can be observed that there were many small holes on the surface of the SiO_2_ disk (Figure 7a). Results following modification of the SiO_2_ disk (Figure 7b) were the same as before modification (Figure 7a). The modification of the SiO_2_ disk with the organosiloxane agent did not affect its permeability. The results show that the modified SiO_2_ disk had the same permeability for H_2_, CO_2_, N_2_ and CH_4_, and were all 1.90 × 10^−6^ mol m^−2^ s^−1^ Pa^−1^. Thus, the modified base without Cu_3_(BTC)_2_ membrane was not selective for H_2_, CO_2_, N_2_ and CH_4_. The obtained Cu_3_(BTC)_2_ membrane was composed of numerous octahedron crystals inlaid and stacked to form a uniform and dense continuous defect-free membrane structure. The scale of the microscope was 200 µm, and the membrane was continuously dense. When the scale was made gradually smaller/the magnification was gradually increased, the positive octahedral structure of the Cu_3_(BTC)_2_ membrane became increasingly obvious but the Cu_3_(BTC)_2_ membrane was composed of several positive octahedral crystals intercalated and stacked to form a uniformly dense continuous defect-free membrane.

### 3.6. The Gas Separation Test of Cu_3_(BTC)_2_ Membrane

The permeability of the modified porous SiO_2_ disk-supported Cu_3_(BTC)_2_ membrane to four components of H_2_, N_2_, CO_2_ and CH_4_ was evaluated. The dynamic diameter of the four gas molecules of H_2_, CO_2_, N_2_ and CH_4_ and the specific results of the membrane permeability to these four gases are listed in Table 1.

The separation experiments of the H_2_/CO_2_, H_2_/CH_4_ and H_2_/N_2_ mixed gases by Cu_3_(BTC)_2_ membrane were conducted to investigate the two-component gas separation of the modified porous SiO_2_ disk-supported Cu_3_(BTC)_2_ membrane. Separation test results of various mixed gases at 298 K and 0.1 MPa are shown in Table 2. These are test results of single component permeable flow and test results of two-component permeable flow. The separation factor and the ideal separation factor calculated according to the results are also shown in Table 2.

### 3.7. Mechanical Stability of Cu_3_(BTC)_2_ Membrane

**Figure 8:**
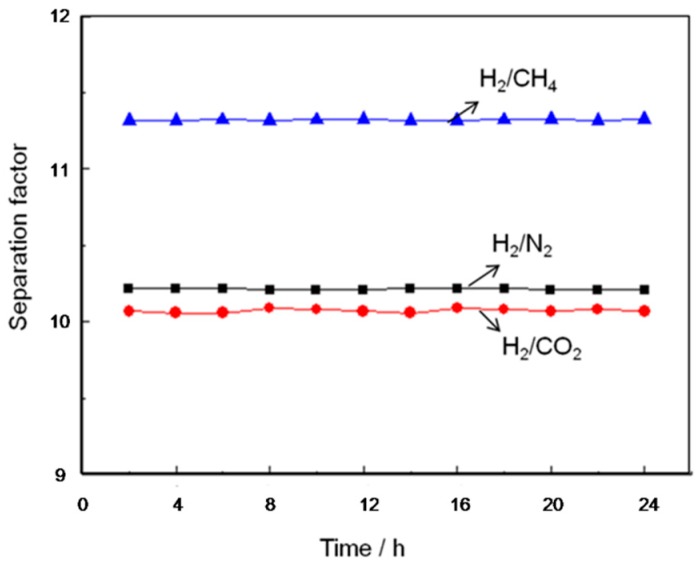
The separation factor of the Cu_3_(BTC)_2_ membrane change with the time: H_2_/CO_2_ (red), H_2_/N_2_ (black), H_2_/CH_4_ (blue).

To study the mechanical properties of the synthesized Cu_3_(BTC)_2_ membrane, the gas separation performance of H_2_/CO_2_ (red), H_2_/N_2_ (black), H_2_/CH_4_ (blue) with the Cu_3_(BTC)_2_ membrane was tested repeatedly under 298 K and 0.1 MPa. Among them, red is powder, black is membrane, and blue is methane. The results show that the separation factor of the Cu_3_(BTC)_2_ membrane, the synthesized membrane reproducibility, was not obviously changed after 24 h of repeated tests. The mechanical properties were strong, and the utilization rate was high (Figure 8).

## 4. Discussion

### 4.1. Preparation of the MOF Membrane

Here, we report a convenient and universal method to prepare MOF membranes by hydrothermal method. First, the porous SiO_2_ disk is soaked in the mixed solution of the concentrated sulfuric acid and the hydrogen peroxide with a volume ratio of 6:4 for 5 h, which gets hydroxyl on the surface of the completely oxidized porous SiO_2_ disk. Then the oxidized porous SiO_2_ disk was modified by N-[3-(Trimethoxysilyl)propyl]ethylenediamine. The FTIR spectra of the modified porous SiO_2_ disk demonstrated that many amino groups existed on the surface of the modified porous SiO_2_ disk, which could be used to grow MOF membrane. The MOF membrane used was Cu_3_(BTC)_2_. The stable 3D structure of the Cu_3_(BTC)_2_ was formed by these secondary structural units interlaced with each other, and the 3D structure had a square aperture with a regular aperture of about 1 nm. The results of N_2_ adsorption show that the specific surface area of BET was about 1550 m^2^/g [42]. The XRD illustrates that the modified porous SiO_2_ disk-supported Cu_3_(BTC)_2_ membrane was a pure phase composed of Cu_3_(BTC)_2_ crystals (Figure 2).

### 4.2. The Morphology and the Stability of the Cu_3_(BTC)_2_ Membrane

The TGA was conducted to investigate the thermal stability of the modified porous SiO_2_disk-supported Cu_3_(BTC)_2_ membrane. The results show that the modified porous SiO_2_ disk-supported Cu_3_(BTC)_2_ membrane was at 63 °C with a weight loss of 5% (the adsorbed water), and the Cu_3_(BTC)_2_ membrane was stable in the air to 300 °C, showing its good thermal stability. The thermogravimetric curve is shown in Figure 5. The characterizations of the morphology of the SiO_2_ disk and the modified porous SiO_2_ disk-supported Cu_3_(BTC)_2_ membrane was conducted after defining the structural information and thermal stability of this membrane. The SEM of the modified porous SiO_2_ disk-supported Cu_3_(BTC)_2_ membrane is shown in Figure 7. It can be observed that the modified porous SiO_2_ disk-supported Cu_3_(BTC)_2_ membrane was a thin, compact, and continuous membrane, closely attached to the modified SiO_2_ substrate. Viewed through the scanning electron microscope, the modified porous SiO_2_ disk-supported Cu_3_(BTC)_2_ membrane also showed that the intergrowth-crystallized octahedral architectures merged tightly. To study the mechanical properties of the synthesized Cu_3_(BTC)_2_ membrane, the gas separation performance of the Cu_3_(BTC)_2_ membrane was tested repeatedly under 298 K and 0.1 MPa. The results show that the separation factor of the Cu_3_(BTC)_2_ membrane, the synthesized membrane reproducibility, was not obviously changed after 24 h of repeated testing. The mechanical properties were strong, and the utilization rate was high (Figure 8).

### 4.3. The Gas Separation Performance of Cu_3_(BTC)_2_ Membrane

The dynamic diameter of the four gas molecules of H_2_, N_2_, CO_2_ and CH_4_ and the specific results of the membrane permeability to these four gases are listed in Table 1. It can be observed that the order of the flow of the four gases through the modified porous SiO_2_ disk-supported Cu_3_(BTC)_2_ membrane was H_2_ > N_2_ > CH_4_ > CO_2_, and the flow rate was 1.61 × 10^−7^ mol m^−2^ s^−1^ Pa^−1^, 1.84 × 10^−8^ mol m^−2^ s^−1^ Pa^−1^, 1.98 × 10^−8^ mol m^−2^ s^−1^ Pa^−1^ and 1.69 × 10^−8^ mol m^−2^ s^−1^ Pa^−1^, respectively. Based on the ideal separation coefficient formula *a* = *J_A_*/*J_B_,* the ideal separation factor of H_2_/CO_2_, H_2_/N_2_, H_2_/CH_4_ were 9.53, 8.75 and 8.13, respectively. It was higher than the corresponding Knudsen diffusion coefficient (4.69 H_2_/CO_2_, 3.74 H_2_/N_2_, and 2.83 H_2_/CH_4_) and was also much larger than the ideal separation factor that was reported to separate the same gas through the Cu_3_(BTC)_2_ membrane [42]. This preliminarily determines that the Cu_3_(BTC)_2_ membrane synthesized in this study are suitable for the separation of H_2_ in the mixed components of H_2_/CO_2_, H_2_/N_2_, H_2_/CH_4_. Since the pore size of the Cu_3_(BTC)_2_ membrane was about 1 nm, which was bigger than the dynamic diameters of H_2_, CO_2_, N_2_ and CH_4_ molecules (Table 1). Thus, there was no effect on H_2_ by the molecular sieve points from the other gases. The Cu_3_(BTC)_2_ structure contained many Cu elements for the adsorption of CO_2_, N_2_ and CH_4_ gases providing the active site and the Cu_3_(BTC)_2_ membrane by chemical adsorption to gas diffusion the effect of separation, as far as the authors are aware. According to reports in the literature, Cu_3_(BTC)_2_ for CO_2_, N_2_ and CH_4_ adsorption enthalpy is far greater than H_2_, and the preparation of the Cu_3_(BTC)_2_ membrane nitrogen adsorption performance showed the same as the previous preparation of fission material adsorption performance. Thus, in this study, some adsorption properties of the MOF powder could be on behalf of the MOF-related adsorption properties of the membrane [43,44,45,46]. Therefore, the separation of two-component gas is investigated. The separation experiments of the H_2_/CO_2_, H_2_/N_2_, H_2_/CH_4_ mixed gases by Cu_3_(BTC)_2_ membrane was conducted to investigate the two-component gas separation of the modified porous SiO_2_ disk-supported Cu_3_(BTC)_2_ membrane. Separation test results of various mixed gases at 298 K and 0.1 MPa are listed in Table 2. There are test results of single component permeable flow, and two-component permeable flow. The separation factor and the ideal separation factor calculated according to the results are also listed in Table 2. Looking at Table 2, it can be observed that the flow of H_2_ in the mixed gas is 1.61 × 10^−7^ mol m^−2^ s^−1^ Pa^−1^, many times higher than the flow of other gases. This phenomenon illustrates that the modified porous SiO_2_ disk-supported Cu_3_(BTC)_2_ membrane has the function of separating and purifying H_2_ and can be used to separate and purify H_2_ in the mixture of H_2_/CO_2_, H_2_/N_2_, H_2_/CH_4_. The flow of H_2_ in the two-component gas is also much higher than that of the other components, it can be obtained through the calculation of the separation factors of the modified porous SiO_2_ disk-supported Cu_3_(BTC)_2_ membrane for the H_2_/CO_2_, H_2_/N_2_, H_2_/CH_4_ components are 10.07, 10.20 and 11.34 in the condition of 298 K and 0.1 MPa. These values are higher than the corresponding Knudsen values. This phenomenon illustrates that the modified porous SiO_2_ disk-supported Cu_3_(BTC)_2_ membrane can be used in gas separation and has a good performance of gas separation.

## 5. Conclusions

This work is the first report of the synthesis of modified porous SiO_2_ disk-supported Cu_3_(BTC)_2_ membranes. The obtained functioned porous SiO_2_ disk-supported Cu_3_(BTC)_2_ membranes have crystal phases that coincide with Cu_3_(BTC)_2_ crystals with a high thermal stability and intact morphology. Additionally, the performances of the modified porous SiO_2_ disk-supported Cu_3_(BTC)_2_ membrane for the separation of hydrogen and other gases were evaluated and the separation factor of each group of experience was calculated in detail. It was found that the membrane has a good separation performance for hydrogen and can be used in hydrogen recovery in industry. This Cu_3_(BTC)_2_ membrane fabrication method is simple and convenient and can be readily applied to a variety of other material compositions to produce functional membranes with diverse micropore structures, thus opening a host of opportunities for the development of new functional MOFs.

## Figures and Tables

**Table 1:** The permeable flow of the single component gas through the modified porous SiO_2_ disk-supported Cu_3_(BTC)_2_ membrane in 298 K and 0.1 MPa.

Gas	Kinetic Diameter (nm)	Permeance (mol m^−2^ s^−1^ Pa^−1^)
H_2_	0.29	1.61 × 10^−7^
CO_2_	0.33	1.69 × 10^−8^
N_2_	0.36	1.84 × 10^−8^
CH_4_	0.38	1.98 × 10^−8^

**Table 2:** The permeable flow of the single component gas and mixed component gas through the modified porous SiO_2_ disk-supported Cu_3_(BTC)_2_ membrane and the separation factor in 298 K and 0.1 MPa.

Gas	Single Component Flow in Mixed Gas (10^−6^ mol m^−2^ s^−1^ Pa^−1^)	Single Component Flow (10^−6^ mol m^−2^ s^−1^ Pa^−1^)	Separation Factor	Ideal Separation Factor
H_2_	0.148	0.161	10.20	8.75
N_2_	0.0145	0.0184		
H_2_	0.152	0.161	11.34	8.13
CH_4_	0.0134	0.0198		
H_2_	0.143	0.161	10.07	9.53
CO_2_	0.0142	0.0169		
